# Stability and Change in Adolescents’ Sense of Agency: Contributions of Sex, Multiple Risk, Pandemic Stress, and Attachment to Parents

**DOI:** 10.1007/s10964-023-01766-x

**Published:** 2023-03-24

**Authors:** Filipa Nunes, Catarina P. Mota, Tiago Ferreira, Ingrid Schoon, Paula M. Matos

**Affiliations:** 1 Faculty of Psychology and Education Sciences, University of Porto. R. Alfredo, Allen, 4200-135 Porto, Portugal; 2 Center for Psychology at University of Porto. R. Alfredo Allen, 4200-135 Porto, Portugal; 3 Escola de Ciências Humanas e Sociais, University of Trás-os-Montes and Alto Douro, Quinta dos Prados, Polo I—UTAD, 5000-801 Vila Real, Portugal; 4 Social Research Institute, University College London (UCL), 55-56 Gordon Square, London, WC1H 0AL UK

**Keywords:** Sense of agency, Latent growth curve, Adolescence, Attachment to parents, Cumulative risk, Pandemic-related stress

## Abstract

Although literature states that individual, relational, and contextual factors contribute to adolescents’ sense of agency, more research is needed to clarify and understand how adolescents develop this belief over time. The current study examined the stability/change trajectories of the sense of agency during adolescence, specifically across high school, analyzing whether attachment to parents over time, adolescents’ sex, cumulative risk in baseline, and pandemic-related stress explained these trajectories. The sample included 467 Portuguese adolescents (40.7% were males; *M*age = 15.58 years, *SD* = 0.80), evaluated three times across 18 months. This work yielded three significant findings. First, adolescents’ sense of agency significantly increased over time, with significant between-subject variance at the initial levels but not at the growth rate. Second, attachment to parents consistently links to adolescents’ sense of agency across time, despite the differential contributions from attachment to mothers and fathers. Third, boys reported greater growth in the sense of agency than girls. Adolescents’ cumulative risk at T1 predicted lower initial levels of sense of agency, whereas higher pandemic-related stress predicted less growth of the sense of agency. These findings emphasize the contributions of individual and family characteristics and the role of the broader social context in shaping the development of adolescents’ sense of agency. The findings underline the need to consider further the differential influences of adolescents’ relationships with mothers and fathers to understand changes in adolescents’ sense of agency.

## Introduction

The sense of agency is a process through which individuals believe they are active agents in directing their life course (Schoon & Cook, 2021). This process translates into the perception of the ability to optimize resources, transform, or overcome constraints to achieve self-determined goals (Zimmerman & Cleary, 2006). Although research on sense of agency has increased in recent decades, it has faced some difficulties arising from different terminology and measurement approaches (Schoon & Cook, 2021). Nonetheless, there is general agreement that the sense of agency is a multidimensional construct (Bandura, 2006; Eccles & Wigfield, 2002; Schoon & Heckhausen, 2019). Its assessment can either focus on specific domains (such as educational attainment) or based on general belief orientations (Hitlin & Johnson, 2015). The current study considered four general facets of sense of agency: goal-setting, optimism, decision-making, and self-efficacy (Nunes et al., 2022b).

Developing sense of agency is essential during adolescence (Zimmerman & Cleary, 2006). Empirical evidence has highlighted the importance of the sense of agency for individuals’ psychoemotional development and adjustment (Gallagher et al., 2019). Nevertheless, the literature on developing an evolving sense of agency during adolescence is scarce. The socio-ecological developmental theories of motivation (Conger et al., 2010; Schoon & Heckhausen, 2019) guided the current study, which sought to fill three gaps in knowledge. First, the current study analyzes the developmental trajectories of sense of agency during late adolescence, specifically across high school and during the significant health pandemic Covid-19. Second, it examines potential differences regarding the role of attachment to mothers and fathers in shaping the development of the sense of agency over time. Third, it considers individual and contextual factors, such as variations by sex, cumulative risk, and pandemic-related stress.

### The Development of Sense of Agency During Adolescence

Sense of agency is not a personality characteristic that remains stable throughout life (Schoon, 2018). Instead, sense of agency development is a dynamic, relational process that develops and evolves from the interactions between the developing individual and a changing social context. Changes in the sense of agency depend on the maturity of the individual, the relational changes, and contextual factors (Schoon & Heckhausen, 2019). Therefore, the sense of agency is malleable through experience (Schoon, 2018). During adolescence, young people develop the ability to make independent decisions. Adolescents gradually become more able to delay gratification and develop an increased concern for their futures. Adolescents go through reorganizations in relationships with their parents and dedicate more time to their peers and romantic partners (Branje, 2018). Thus, late adolescence may be a promising period for detecting changes in sense of agency.

Young people face developmental challenges during adolescence as the construction of personal identity, the progressive acquisition of autonomy, planning for the future, and abstract thinking development. Facing such tasks will allow young people to progressively acquire a greater capacity for self-regulation (Branje, 2018), a central aspect of the sense of agency. Note that sense of agency means, in its essence, “believing to be the author of own voluntary behavior” (Deci & Ryan, 2004). Further, cognitive advances experienced during adolescence can lead young people to gain greater decision-making power in contexts controlled by their parents or teachers at earlier ages. Thus, as adolescents develop their cognitive and emotional competencies, they may experience greater ease in setting goals based on their values. They also can reveal a more optimistic perspective about the future, feel greater power to make decisions, and believe strongly in their ability to achieve volitional goals (Conger et al., 2010).

The need to adapt to new educational contexts resulting from the transition to high school is a significant change that young people face across late adolescence. In Portugal, high school starts in the 10th grade (14/15 years), requiring students to make important decisions about the future (Torres & Mouraz, 2019). Upon entering high school, young people make vocational choices, which can be a significant step toward their affirmation. Also, young people are given more power during high school to plan and organize their learning tasks. Their investments are made to a specific goal (higher education or labor market), and they progressively achieve greater decision-making power regarding their future, which can increase their sense of agency.

Another change young people experience during adolescence is attachment reorganization, i.e., the changes in relationships with parents. During adolescence, young people show progressively greater facility in distancing themselves from their parents’ ideas and choosing according to their volitional values (Allen, 2004; Branje, 2018). Adolescents increasingly become active agents in their widening social world, striving to develop their sense of agency as they assert their place in the family and their autonomy from their parents (Thoits, 2006). A more in-depth reflection on this issue will be addressed below.

In the Portuguese cultural context, young people reach the age of majority at 18 years old—i.e., youth become legally responsible for their actions, also having total civil obligations and rights. Therefore, at this stage of their lives, young Portuguese are expected to become more autonomous and independent—this cultural expectation might boost the youth’s sense of agency. Even so, Portuguese youth at this age only sometimes achieve complete independence from their parents. The postponement of independence from family mainly results from sociocultural changes, including youth increased investment in longer educational trajectories and subsequent delay of professional integration, as well as increasing economic instability and labor uncertainty (Mota et al., 2022; Saraiva & Matos, 2016).

Based on cognitive, emotional, and relational changes that young people experience during adolescence and the importance that these changes have for their sense of agency (Eccles & Wigfield, 2002), the average levels of sense of agency could increase during adolescence. Previous empirical evidence also shows that some indicators of a sense of agency increased during adolescence (Gutman & Eccles, 2007; Wray-Lake et al., 2010). For instance, in a 6-year longitudinal study of 1329 adolescents (from 13 to 19 years), the decision-making power increased over time (Gutman & Eccles, 2007). A higher increase occurred between ages 15 and 17 (during high school). This finding was attributed to a progressive match between the needs of developing adolescents and the opportunities in their various social contexts (Eccles et al., 1993). Another longitudinal study analyzed the patterns in parents’ reports of 201 families about the decision-making autonomy of their children between ages 9 to 20 years. Decision-making autonomy increased gradually across middle childhood and adolescence before rising sharply in late adolescence, after age 15 (Wray-Lake et al., 2010). These results are consistent with the person-environment adaptation perspective (Eccles et al., 1993), which considers that an ideal family context should provide higher levels of control and lower levels of autonomy in early childhood and the reverse pattern in late adolescence.

### Attachment to Parents and Sense of Agency

Humans have an inherent ability to create and develop emotional bonds with significant others. According to attachment theory-guided (Ainsworth, 1969; Bowlby, 1988) empirical studies, one’s secure relationships with caregivers are characterized by trust in their availability and responsiveness (Bowlby, 1988) and are linked to the development of more positive dynamic internal models (Lopez & Brennan, 2000). These positive models about themselves, others, and the world facilitate the individual’s development of self-regulation (Allen & Miga, 2010) and autonomy (McElhaney et al., 2009).

The period of adolescence is especially characterized by rapid biopsychosocial changes that will impact the adolescent’s relationships with others. For example, parents and adolescents are likely to reorganize their interactions to promote their experience of more egalitarian and reciprocal relationships with one another (Branje, 2018), which promotes the adolescents’ biological or cognitive maturation and predicts developmental changes in parent-adolescent relationships (Allen, 2004). Hormonal changes related to puberty can lead adolescents to seek autonomy and initiate separation-individuation from parents. These relational changes do not imply that parents lose their relevance as attachment figures but that adolescents become less dependent on them (Allen, 2004). Parents are likely to remain a secure base for adolescents when necessary (Allen, 2004) and can shape adolescents’ sense of agency (Nunes et al., 2022a).

Given the relational nature of the sense of agency, it is important to consider the contributions of adolescents’ attachment to parents when analyzing their development of a sense of agency over time. Attachment to parents may have a stable contribution to the adolescent’s sense of agency over time. On the other hand, specific attachment dimensions to parents may have a more time-specific effect on the adolescent’s sense of agency. In addition, as the relative roles of mothers and fathers in shaping the adolescent’s development of the sense of agency over time remain unclear (Schoon & Eccles, 2014), the current study will consider the role of both parents in this process. Although it is important to recognize that adaptive changes and reorganizations in adolescence are also shaped by peers (Allen et al., 2007; Schoon, 2018), the current study focuses exclusively on the adolescent’s attachment to each parent.

### Individual and Contextual Factors: The role of Sex, Cumulative Risk, and Pandemic-Related Stress in Shaping Sense of Agency

Socio-ecological models of human development emphasize the role of multiple contextual factors in shaping the sense of agency (Conger et al., 2010), including the contribution of sex, cumulative risk exposure, and characteristics of changing social context. There is established evidence of sex differences in adolescents’ sense of agency, with boys reporting a stronger sense of agency than girls (Schoon & Cook, 2021). Gender stereotypes and inequalities in most Western societies can explain these findings (Schoon & Eccles, 2014).

The accumulation of risk situations can enhance adolescents’ perception that they have limited resources to face, modify or overcome life’s constraints (Conger et al., 2010). The current study focuses on psychosocial risk, defined as the absence of development opportunities resulting from poor contextual experiences unsuited to individuals’ needs (World Health Organization, 2020). According to the literature, features of the family’s immediate context, such as the size of the household (single-parent families or large families with three minors), the parents’ low education and the disqualified professional occupations, as well as low family income, tend to impoverish individuals’ developmental experiences (Gutman et al., 2019). Several studies frame these features as risk factors for youth development (e.g., Guedes, 2015; Gutman et al., 2019; Price & Hyde, 2009; Schoon & Henseke, 2022). For instance, low parental education and unskilled professional occupations are associated with a family’s lower income and children’s health problems (Buehler and Gerard 2013; January et al., 2017). Unemployment and parental retirement (accompanied by financial difficulties) can limit the options and, consequently, young people’s choices (Guedes, 2015; Sleskova et al., 2006). These situations tend to diminish resources and promote adaptation problems. Retirement can give individuals more time to dedicate to personal and family activities. However, when the individual does not voluntarily choose to retire, this transition might be the source of physical and mental health issues and significant economic concerns, with implications for adolescents’ well-being (Dave et al., 2008). Other risk factors described in the literature include experiencing adverse life events, such as bereavement (e.g., Harrison & Harrington, 2001), parental separation/divorce (e.g., Obeid et al., 2021) and academic or residential mobility (e.g., Langenkamp, 2016; Simsek et al., 2021). Due to these factors, young people may feel more constrained to take risks and explore new paths (Schoon & Lyons-Amos, 2017).

Despite the importance of individual risk factors, the current study focuses on the accumulation of different risk factors, which determines the context’s adversity (Gutman et al., 2002). Several empirical studies have supported this assumption, showing that the number of risk factors is more relevant for determining the developmental outcome than any single factor considered alone (e.g., Dannefer & Huang, 2017; Price & Hyde, 2009). Based on this notion, the most common approach for measuring a context’s adversity is to add up a set of dichotomized risk factors (1 = risk; 0 = no risk) into a cumulative risk index (Gutman et al., 2019). Given the higher developmental challenges that young people face, adolescence tends to be a period of some emotional lability that may be exacerbated by the experience of cumulative risk situations. From this perspective, unraveling the contribution of cumulative risk to the adolescent sense of agency development is relevant.

Furthermore, it was considered the role of broader contextual factors, such as challenges due to the Covid-19 pandemic, which disproportionately affected young people (Cohen et al., 2021). In addition to its physical health consequences, the Covid-19 pandemic substantially impacted the mental health of individuals, particularly adolescents (Cohen et al., 2021; Kowal et al., 2020), as well as their education, training and employment opportunities (ILO, 2020; Harmey & Moss, 2021). Many aspects of the pandemic, such as fear of infection, general lockdown, social isolation, and distance learning, likely elevated stress reactions that could undermine adolescents’ mental health (Duan et al., 2020). Evidence also suggests that the COVID-19 pandemic can adversely impact adolescents’ sense of agency and outlook on the future (Schoon & Cook, 2021). The current study will consider and analyze the contribution of pandemic-related stress on the evolution of the sense of agency during adolescence. The contribution of pandemic-related stress on initial values of the sense of agency will not be examined as the first wave of data collection occurred before the COVID-19 outbreak.

## Current Study

Although the literature states that the sense of agency is not a personality trait and can reveal changes, there is little evidence regarding its development during adolescence. The current study provides three main contributions to addressing this knowledge gap. First, it examines the stability and change in the sense of agency across late adolescence, specifically across high school and during the Covid-19 pandemic. It was expected growth in the average levels of sense of agency over time (Hypothesis 1). Second, this study investigates whether variations in attachment to each parent are related to the trajectory of adolescents’ sense of agency. It was anticipated that attachment to mothers and fathers relates to adolescents’ sense of agency differentially (Hypothesis 2). Third, the current study analyzes variations in initial levels and the trajectory of sense of agency by adolescents’ sex and exposure to cumulative risk, controlling the school year adolescents attended at baseline. It was expected that girls and those exposed to cumulative risk would report lower levels of sense of agency (Hypotheses 3 and 4). However, there is less certainty regarding the links between these factors’ developmental trajectory of adolescents’ sense of agency over time. It also examined the contribution of pandemic-related stress on the trajectory of sense of agency. In particular, it was hypothesized that pandemic-related stress would undermine agency development (Hypothesis 5).

## Methods

### Participants

The sample includes 467 adolescents in T1 (59.3% were girls; *Mage* = 15.58 years, *SD* = 0.80), who were evaluated three times across 18 months. The baseline assessment (T1) occurred between September and December 2019. After 12 months of initial assessment, adolescents responded again to protocol (T2). The last assessment (T3) occurred six months after the second assessment. Adolescents’ mean age was 16.52 at T2 (*SD* = 0.76) and 16.93 at T3 (*SD* = 0.87). Most of the participants (78.6%) lived with both parents, and some were living only with their mother (18.4%) or father (3.0%). There were 0.6% of missing values. Fifty-two-point three percent of adolescents were in the 10^th^ grade, and 47.3% were in the 11^th^ grade. This level of education corresponds to level three of the International Standard Classification of Education (ISCED) (UNESCO, 2011).

Eighty-three adolescents had missing data at T2 (17.77%), and 117 adolescents had missing data at T3 (25.05%). The attrition rate was 12.21% (*n* = 57) at T2, and 12.85% (*n* = 60) at T3. The attrition in T2 was mostly due to the wrong inclusion of a class in T1 that would not attend the same school in the following school year (after 12 months). This class was wrongly selected at T1 by the school board to participate in the current study. Attrition at T3 was mainly due to the misidentification of two classes by the research team. It was impossible to pair the observation of these students at T2 with their reports in T3. Results from Little’s MCAR tests (Little, 1988) indicate that the observed patterns of missing data were not consistent with the assumption of missing completely at random (MCAR) (χ^2^_(342)_ = 473.63, *p* = 0.001). Several univariate regression models analyzed the missing data mechanism to clarify the associations between individual and family characteristics at T1 (sex, family income, residence, parents’ marital status) and adolescents’ participation status in T2 and T3. The results showed that the attrition was mainly due to adolescents’ sex and that boys were likelier to drop out of the study than girls (χ^2^_(1466)_ = 14.12, *p* = 0.001). According to Little and Rubin (2002), if the missing data are related to measured variables but not to an unmeasured variable, these data can be assumed to be Missing at Random (MAR). Given the significant contribution of the adolescents’ sex to attrition, the missing data were assumed MAR. Therefore, the full information maximum likelihood (FIML) was used for data analysis.

### Measures

#### Sociodemographic measure

Adolescents completed a brief questionnaire about their data, such as age, sex, school year, household, and parents’ marital status.

#### Sense of agency

In the three assessment points, adolescents responded to an empirical model of a sense of agency that combined four dimensions: goal-setting, decision-making, optimism, and self-efficacy (Nunes et al., 2022b). Adolescents answered of goal-setting (GS) and decision-making (DM) dimensions of the Short Self-Regulation Questionnaire (SSRQ; Carey et al., 2004; Portuguese version by García Del Castillo & Dias, 2009). The GS subscale assesses the ability to plan and set clear goals (seven items; e.g., “When I have a goal, I usually plan how to achieve it”). The DM subscale assesses the ability to engage in decision-making processes (five items; e.g., “I put off making decisions”). The SSRQ’s items are completed using a five-point scale, ranging from (1) “strongly disagree” to (5) “strongly agree”. Both dimensions revealed adequate consistency (α = 0.75/0.80/0.86 for GS; α = 0.69/0.75/0.82 for DM) in T1, T2 and T3, respectively. Adolescents also responded to the optimism subscale of Vision About Future (VAF; Ginevra et al., 2016, Portuguese version by Nunes et al., 2018). This subscale assesses the individual’s orientation toward expecting general positive results in the future (seven items; e.g., “Usually, I am full of enthusiasm and optimism about my future”). Items are rated on a five-point scale ranging from (1) “*it does not describe me at all*” to (5) “*it describes me very well*”. The optimism subscale revealed good consistency (α = 0.89/0.89/0.91) in T1, T2 and T3, respectively. Further, adolescents completed the General Self-Efficacy Scale (GSE; Schwarzer & Jerusalem, 1995, Portuguese version by Nunes et al., 1999), which evaluated their self-efficacy. This unidimensional scale assesses beliefs concerning one’s self-capacity to deal with demands and problems (10 items; e.g., “I can solve most problems if I invest the necessary effort”). The items of GSE are rated on a four-point scale ranging from (1) “*not at all true*” to (4) “*exactly true*”. Scores on the self-efficacy dimension demonstrated adequate consistency (α = 0.73/0.80/0.84) at each of the study’s three assessment time points. The model combining the four indicators showed adequate reliability (α = 0.88/0.90/0.92) at the study’s three assessment time points. The response scale of the self-efficacy dimension (ranging from 1 to 4) was converted to a 5-point scale (ranging from 1 to 5). So, all dimensions of the sense of agency were expressed in the same measuring scale.

#### Attachment to parents

Participants completed the Quality of the Emotional Bond (QEB) and Inhibition of Exploration and Individuality (IEI) subscales of the Father and Mother Attachment Questionnaire – short form (FMAQ; Nunes et al., 2020a). The QEB subscale assesses the individual’s importance on parents as attachment figures (five items: e.g., “I rely on my parents’ support in difficult moments of my life.”). The IEI subscale measures the individuals’ perception that their parents are actively constraining and discouraging their individuality and exploratory movement (five items; e.g., “At home, it is a problem when my interests differ from my parents.”). The responses are rated on a six-point scale ranging from (1) “*totally disagree*” to (6) “*totally agree*”. The internal consistency analysis yielded the following values on the father version of these subscales across the three assessment time points: QEB = 0.86/0.90/0.92, and IEI = 0.66/0.74/0.84, whereas the following values on the mother version of these subscales over these same time points: QEB = 0.86/0.88/0.91, and IEI = 0.65/0.73/0.80. FMAQ scores were compared to the results from a semi-structured interview protocol administered to 82 adolescents (Family Attachment Interview; Bartholomew & Horowitz, 1991) and coded by independent judges, revealing small to moderate correlations. Evidence for the convergent validity of the FMAQ scores was also obtained (Gouveia & Matos, 2013). QBE and IEI dimensions showed moderate to high correlations with subscales of another widely used attachment measure, the Inventory of Parent and Peer Attachment (IPPA, Armsden & Greenberg, 1987; PBI, Parker et al., 1979).

#### Cumulative risk

Participants completed a Multi-Risk Questionnaire concerning their exposure to a range of psychosocial risks identified in the literature as relevant to the development of agency (Gutman et al., 2002; Johnson & Hitlin, 2017; Spisma et al., 2015), including:

##### Not live with both parents

Adolescents who do not live with both parents. Scores: Zero—No Risk, One—Risk.

##### Low parental education

Education equal to or lower than the 6^th^ grade. Scores: Zero—No Risk; One—Risk associated with one parent; Two—Risk associated with both parents.

##### Unskilled parental occupations

Unemployment, retirement, and unskilled work. Zero—No Risk; One—Risk associated with one parent; Two—Risk associated with both parents.

##### Low family income

Families with incomes below the minimum wage. Scores: Zero—No Risk, One—Risk.

##### Change of residence or school (experienced by adolescents)

Change of residence or school within the last five years. Scores: Zero—No Risk; One—Risk.

##### Accident or severe illness (experienced by adolescents)

The experience of an accident or serious illness in the last five years. Scores: Zero—No Risk, One—Risk.

##### Experience two or more negative events (experienced by adolescents)

The experience of two or more negative events (e.g., death of a close relative, divorce, or separation from parents) in the last five years. Scores: Zero—No Risk, One—Risk.

The last three situations (change of residence or school; accident or severe illness; experience of two or more negative events) were only considered a risk if adolescents reported that the experience harmed their lives. The impact was analyzed through a Likert scale ranging from (0) “*did not affect me negatively*”; to (4) “*It affected me a lot*”). Scores of two, three, and four indicate that the situations reported in the last three risk factors harmed adolescents’ lives. Guided by cumulative risk model assumptions (Conger et al., 2010; Gutman et al., 2002), a composite risk index (CRI) was calculated by the sum of the scores obtained on each of the seven risk factors. CRI varies on a scale from zero to nine. A lower score indicates low-risk accumulation. Approximately 41.5% of adolescents experienced four or more multiple risks. The risk factor with a higher incidence among participants was the unskilled parental occupation associated with both parents (64.7%) (Table 1).

**Table 1:** Incidence of risk factors

**Risk factors**	**Incidence**
Both parents in unskilled occupations	64.7%
Recent negatively felt experience of two or more negative events	42.6%
Low education of one parent	24.2%
Negatively experienced change of residence or school	23.1%
Low education of both parents	22.3%
One parent in an unskilled occupation	21.8%
Live with only one parent	21.4%
Low family income	13.1%
Recent negatively felt experience of an accident or serious illness	12.4%
**Composite Risk Index (CRI)**	**Incidence**
0—no risk experiences	3.4%
1—one risk experience	6.4%
2—two risk experiences	15.6%
3—three risk experiences	16.5%
4—four risk experiences	20.1%
5—five risk experiences	13.5%
6—six risk experiences	4.9%
7—seven risk experiences	2.4%
8—eight risk experiences	0.6
9—nine risk experiences	0%
Missing	16.5%

#### Pandemic-related stress

Given the absence of a measure to assess the impact of COVID-19, an instrument widely used in the scientific community regarding perceived stress was adapted to the pandemic situation. Adolescents completed an adapted version of the perceived distress dimension of the Perceived Stress Scale (PSS-10; Cohen et al., 1983, Portuguese version by Amaral et al., 2015) to COVID-19 pandemic situation (six items; e.g., “During the covid-19 pandemic, I felt more nervous or stressed”) (Nunes et al., 2020b). The responses are given on a five-point scale, ranging from (0) “*never*” to (4) “*very frequent*”. This dimension was assessed in T2 and presented adequate consistency (α = 0.74).

### Procedures

Data were collected within a broader research project aiming to understand the impact of the individual, relational, family, and school factors on the development of the sense of agency of adolescents. Authorizations were obtained from the Ethics Committee of the Faculty of Psychology and Education Science at the University of Porto, the data protection officer at the University of Porto, and the Portuguese Ministry of Education to administer the questionnaires in the school context. First, all public schools (*N* = 71) of the district of Porto, Portugal, were contacted by phone and email. Meetings with the directors’ boards that responded to the initial requests of the research team were conducted. Eight schools agreed to participate in the current study, which comprises three assessment times. The research project was brief present to students. Parents signed written informed consent, while adolescents signed written informed assent. The research planned to include three assessments six months apart. With the outbreak of the pandemic and the consequent closing of schools, the assessment points were redefined. T2 data was collected 12 months after T1, while T3 data were collected six months after T2. Data collection at T2 and T3 also occurred in classrooms during standard school hours. However, due to covid-19 restrictions, the research team could only provide online support during these data collections. Adolescents who agreed to participate completed a questionnaire on attachment to each parent and sense of agency at T1, T2, and T3. Adolescents also reported multiple psychosocial risks in T1 and their distress due to COVID-19 at T2. The adolescents did not receive any reward for participation.

### Data Analysis

The Z score and the Mahalanobis distance identified univariate and multivariate outliers. The statistical analysis did not consider participants identified as outliers (*n* = 6). Adolescents’ data did not show severe deviations from normality (Kline, 2015). Means, standard deviation, and correlations among variables were calculated. The factorial structure of measures was also tested through confirmatory factor analysis (CFA). The following cut-offs guided the interpretation of CFA results: CFI and TLI ≥ 0.80, RMSEA and SRMR < 0.10 to indicate an acceptable fit (Kline, 2015). Further, the longitudinal invariance of the sense of agency and attachment to parents was tested. The invariance of attachment to parents among parents’ sex was also tested. The following cut-offs guided the invariance results interpretation: nonsignificant Δχ^2^, ΔCFI ≤ 0.010, and ΔRMSEA < 0.015. According to Cheung and Lau (2012), even though Δχ^2^ is significant if ΔCFI and ΔRMSEA are within these cut-off points, this means that differences between the models are tiny and that invariance of the more restricted model can be assumed. The results of intraclass correlations (ICCs) indicated that a low proportion of variance in adolescents’ sense of agency was related to the class (ICC = 0.07/0.12/0.06) and school levels (ICC = 0.04/0.01/0.01) in T1, T2, and T3. A design-based estimation approach corrected standard errors for potential nonindependence of observations (Muthén & Satorra, 1995). An unconditional latent growth curve (LGC) was conducted with latent variables analyses using structural equation modeling. The LGC model was tested using the saturated correlated approach, i.e., adolescents’ sex as introduced in the model as an auxiliary variable (Newson, 2015). Finally, several conditional LGC models examined the contributions of attachment to parents on adolescents’ sense of agency over time and the differences or similarities in the individual contribution of attachment to father and mother. Further, these models analyzed the role of sex and multiple psychosocial risks at baseline and pandemic-related stress at T2 on the initial values and change of sense of agency. A robust maximum likelihood estimation corrected the non-normality and nonindependence of data. Also, under the assumption of MAR, full information maximum likelihood estimation was used to avoid deleting subjects with missing data (Enders & Bandalos, 2001). All analyses were conducted in R (R Core Team, 2020), using the *lavaan* (Rosseel, 2012) and the *semTools* packages (Jorgensen, 2019).

## Results

### Preliminary Analyses: Confirmatory Factor Analyses and Measurement Invariance

The model combining the four indicators of sense of agency (goal-setting, decision-making, optimism, and self-efficacy) showed an acceptable fit at T1, T2, and T3. The attachment questionnaire demonstrated a good fit for fathers’ and mothers’ versions at T1, T2, and T3. The perceived distress related to COVID-19 revealed an acceptable fit at T2 (Table 2). The measurement invariance results revealed that the sense of agency is invariant over time at the scalar level (Δχ^2^_(8)_ = 23.25, *p* = 0.003, ΔCFI = −0.007; ΔRMSEA = 0.004). Fathers’ version of attachment revealed longitudinal scalar invariance (Δχ^2^_(12)_ = 37.61, *p* = 0.001; ΔCFI = −0.005, ΔRMSEA = 0.000); while mothers’ version revealed longitudinal residual invariance (Δχ^2^_(9)_ = 3.78, *p* = 0.925). Further, metric invariance among parents’ sex for the three assessment points was found (Δχ^2^_(50)_ = 87.05, *p* = 0.001; ΔCFI = −0.005, ΔRMSEA = 0.001).

**Table 2:** Confirmatory factor analyses

Measure	T1	T2	T3
Sense of agency	χ^2^_(182)_ = 575.99, *p* = 0.001, CFI = 0.89, TLI = 0.88, RMSEA = 0.07, SRMR = 0.06	χ^2^_(180)_ = 614.09, *p* = 0.001, CFI = 0.88, TLI = 0.86, RMSEA = 0.07, SRMR = 0.07	χ^2^_(180)_ = 537.45, *p* = 0.001, CFI = 0.91, TLI = 0.89, RMSEA = 0.07, SRMR = 0.07
FMAQ FATHER	(χ^2^_(34)_ = 127.92, *p* = 0.001, CFI = 0.94, TLI = 0.92, RMSEA = 0.08, SRMR = 0.04	χ^2^_(34)_ = 92.51, *p* = 0.001, CFI = 0.97, TLI = 0.96, RMSEA = 0.07, SRMR = 0.04	χ^2^_(32)_ = 85.03, *p* = 0.001, CFI = 0.97, TLI = 0.96, RMSEA = 0.07, SRMR = 0.03
FMAQ MOTHER	χ^2^_(34)_ = 77.93, *p* = 0.001, CFI = 0.97, TLI = 0.96, RMSEA = 0.05, SRMR = 0.03	χ^2^_(34)_ = 77.58, *p* = 0.001, CFI = 0.97, TLI = 0.96, RMSEA = 0.06, SRMR = 0.03	χ^2^_(33)_ = 94.66, *p* = 0.001, CFI = 0.97, TLI = 0.96, RMSEA = 0.07 SRMR = 0.03
PSS	NA	χ^2^_(8)_ = 25.68, *p* = 0.001, CFI = 0.97, TLI = 0.94, RMSEA = 0.08, SRMR = 0.04	NA

*FMAQ* *Father*—Attachment to father, *FMAQ Mother*—Attachment to mother, *PSS* Perceived Stress Scale, *NA* Not applicable

### Correlations

Table 3 presents the covariances among the study variables and the means and standard deviations. Indicators of sense of agency positively correlated with the quality of the emotional bond between adolescent and their father and mother. Also, the indicators of sense of agency negatively correlated with the inhibition of exploration and individuality concerning both parents, multiple psychosocial risks, and pandemic stress.

**Table 3:** Covariances over time

	1	2	3	4	5	6	7	8	9	10	11	12	13	14	15	16	17	18	19	20	21	22	23	24	25	26	27
**T1**																											
1. SG	–																										
2. MD	0.33^**^	–																									
3. OPT	0.37^**^	0.38^**^	–																								
4.SE	0.37^**^	0.46^**^	0.55^**^	–																							
5.Q_F	0.29^**^	0.17^**^	0.26^**^	0.15^**^	–																						
6.I_F	−0.22^**^	−0.28^**^	−0.20^**^	−0.15^**^	0.59^**^	–																					
7.Q_M	0.33^**^	0.20^**^	0.29^**^	0.26^**^	0.59^**^	−0.45^**^	–																				
8.I_M	−0.26^**^	−0.24^**^	−0.24^**^	−0.21^**^	−0.42^**^	0.74^**^	−0.59^**^	–																			
9.M_R	0.05	−0.14^**^	−0.10	−0.14^**^	−0.12^**^	0.15^**^	−0.03	0.15^**^	–																		
**T2**																											
10.SG	0.53^**^	0.24^**^	0.32^**^	0.28^**^	0.19^**^	−0.10^**^	0.25^**^	−0.23^**^	−0.06	–																	
11. MD	0.26^**^	0.56^**^	0.38^**^	0.36^**^	0.18^**^	−0.15^**^	0.16^**^	−0.16^**^	0.13^*^	0.41^**^	–																
12. OPT	0.29^**^	0.30^**^	0.64^**^	0.33^**^	0.22^**^	−0.15^**^	0.17^**^	−0.18^**^	−0.16^**^	0.49^**^	0.45^**^	–															
13.SE	0.25^**^	0.36^**^	0.42^**^	0.50^**^	0.14^**^	−0.07	0.15^**^	−0.09	−0.19^**^	0.41^**^	0.44^**^	0.58^**^	–														
14.Q_F	0.30^**^	0.19^**^	0.32^**^	0.15^**^	0.65^**^	−0.44^**^	0.33^**^	−0.29^**^	−0.15^**^	0.23^**^	0.22^**^	0.35^**^	0.21^**^	–													
15.I_F	−0.24^**^	−0.22^**^	−0.20^**^	−0.09	−0.47^**^	0.55^**^	−0.29^**^	0.36^**^	0.08	−0.15^**^	−0.21^**^	−0.24^**^	−0.13^**^	−0.60^**^	–												
16.Q_M	0.65^**^	0.23^**^	0.34^**^	0.26^**^	0.39^**^	−0.33^**^	0.64^**^	−0.48^**^	−0.04	0.32^**^	0.25^**^	0.34^**^	0.26^**^	0.59^**^	−0.42^**^	–											
17.I_M	−0.28^**^	−0.23^**^	−0.16^**^	−0.17^**^	−0.29^**^	0.41^**^	−0.47^**^	0.53^**^	0.04	−0.26^**^	−0.22^**^	−0.23^**^	−0.16^**^	−0.40^**^	0.71^**^	0.67^**^	–										
18. PSS	−0.01	−0.23^**^	−0.21^**^	−0.18^**^	−0.06	0.06	−0.08	0.10^*^	0.13^*^	−0.14^**^	−0.31^**^	−0.29^**^	−0.35^**^	−0.05	0.12^*^	−0.08	0.11^*^	–									
**T3**																											
19.SG	0.50^**^	0.24^**^	0.32^**^	0.24^**^	0.27^**^	−0.−0.14^**^	0.24^**^	−0.22^**^	−0.07	0.69^**^	0.38^**^	0.45^**^	0.31^**^	0.23^**^	−0.21^**^	0.31^**^	−0.27^**^	−0.17^**^	–								
20. MD	0.28^**^	0.54^**^	0.38^**^	0.35^**^	0.17^**^	−0.14^**^	0.16^**^	−0.19^**^	−0.15^**^	0.34^**^	0.65^**^	0.41^**^	0.36^**^	0.22^**^	−0.16^**^	0.26^**^	−0.20^**^	−0.32^**^	0.43^**^	–							
21. OPT	0.24^**^	0.29^**^	0.60^**^	0.34^**^	0.27^**^	−0.17^**^	0.19^**^	−0.20^**^	−0.21^**^	0.41^**^	0.48^**^	0.78^**^	0.48^**^	0.34^**^	−0.26^**^	0.30^**^	−0.23^**^	−0.33^**^	0.51^**^	0.51^**^	–						
22.SE	0.23^**^	0.35^**^	0.40^**^	0.47^**^	0.17^**^	−0.11^*^	0.11^*^	−0.08	−0.18^**^	0.31^**^	0.42^**^	0.50^**^	0.61^**^	0.23^**^	−0.21^**^	0.18^**^	0.17^**^	−0.35^**^	0.41^**^	0.47^**^	0.59^**^	–					
23.Q_F	0.26^**^	0.18^**^	0.27^**^	0.07	0.68^**^	−0.47^**^	0.41^**^	−0.40^**^	−0.17^**^	0.25^**^	0.21^**^	0.34^**^	0.17^**^	0.81^**^	−0.63^**^	0.53^**^	−0.46^**^	−0.15^**^	0.32^**^	0.21^**^	0.38^**^	0.18^**^	–				
24.I_F	−0.16^**^	−0.18^**^	−0.16^**^	−0.04	−0.44^**^	0.53^**^	−0.36^**^	0.44^**^	0.16^**^	−0.16^**^	−0.19^**^	−0.21^**^	−0.08	−0.55^**^	0.69^**^	−0.39^**^	0.50^**^	0.18^**^	−0.26^**^	−0.21^**^	−0.24^**^	−0.16^**^	−0.67^**^	–			
25.Q_M	0.33^**^	0.19^**^	0.31^**^	0.14^**^	0.39^**^	−0.37^**^	0.63^**^	−0.50^**^	−0.08	0.34^**^	0.27^**^	0.32^**^	0.20^**^	0.44^**^	−0.46^**^	0.81^**^	−0.67^**^	−0.12^*^	0.40^**^	0.26^**^	0.34^**^	0.20^**^	0.66^**^	−0.52^**^	–		
26.I_M	−0.20^**^	−0.14^**^	−0.17^**^	−0.08	−0.29^**^	0.40^**^	−0.45^**^	0.53^**^	0.08	−0.24^**^	−0.23^**^	−0.19^**^	−0.08	−0.33^**^	0.47^**^	−0.55^**^	0.65^**^	0.10	−0.34^**^	−0.25^**^	−0.24^**^	−0.13^*^	−0.49^**^	0.81^**^	−0.67^**^	–	
27.Sex	−0.19^**^	0.04	0.13^**^	0.07	0.02	0.14^**^	−0.05	0.13^**^	−0.03	−0.09	0.07	0.10	0.15^**^	−0.01	0.06	−0.08	0.14^**^	−0.31^**^	−0.07	0.12^*^	0.19^**^	0.21^**^	0.05	0.07	−0.03	0.12^*^	–
***Mean***	3.98	3.13	3.36	3.15	5.07	2.06	5.40	2.00	3.52	3.93	3.15	3.29	3.23	4.92	2.15	5.30	2.04	2.07	3.92	3.11	3.31	3.26	4.90	2.20	5.23	2.06	1.41
***SD***	0.55	0.72	0.91	0.83	0.97	0.92	0.77	0.89	1.65	0.55	0.73	0.87	0.78	1.08	1.01	0.85	0.94	0.86	0.64	0.81	0.95	0.82	1.10	1.13	0.91	1.03	0.41

*SG* Setting goals, *MD* Making decision, *OPT* Optimism, *SE* Self-efficacy, *Q_F* Quality of Emotional Bond of the father, *I_F* Inhibition of Exploration and Individuality of the father, *Q_M* Quality of Emotional Bond of the father, *I_M* Inhibition of Exploration and Individuality of mother, *M_ R* Multiple psychosocial risks, *PSS* Stress due to pandemic, *Sex* Adolescents’ sex, *SD* Standard deviation

*p < 0.05, two-tailed. **p < 0.01, two-tailed

### Latent Growth Curve (LGC): Unconditional and Conditional Model

**Fig. 1:**
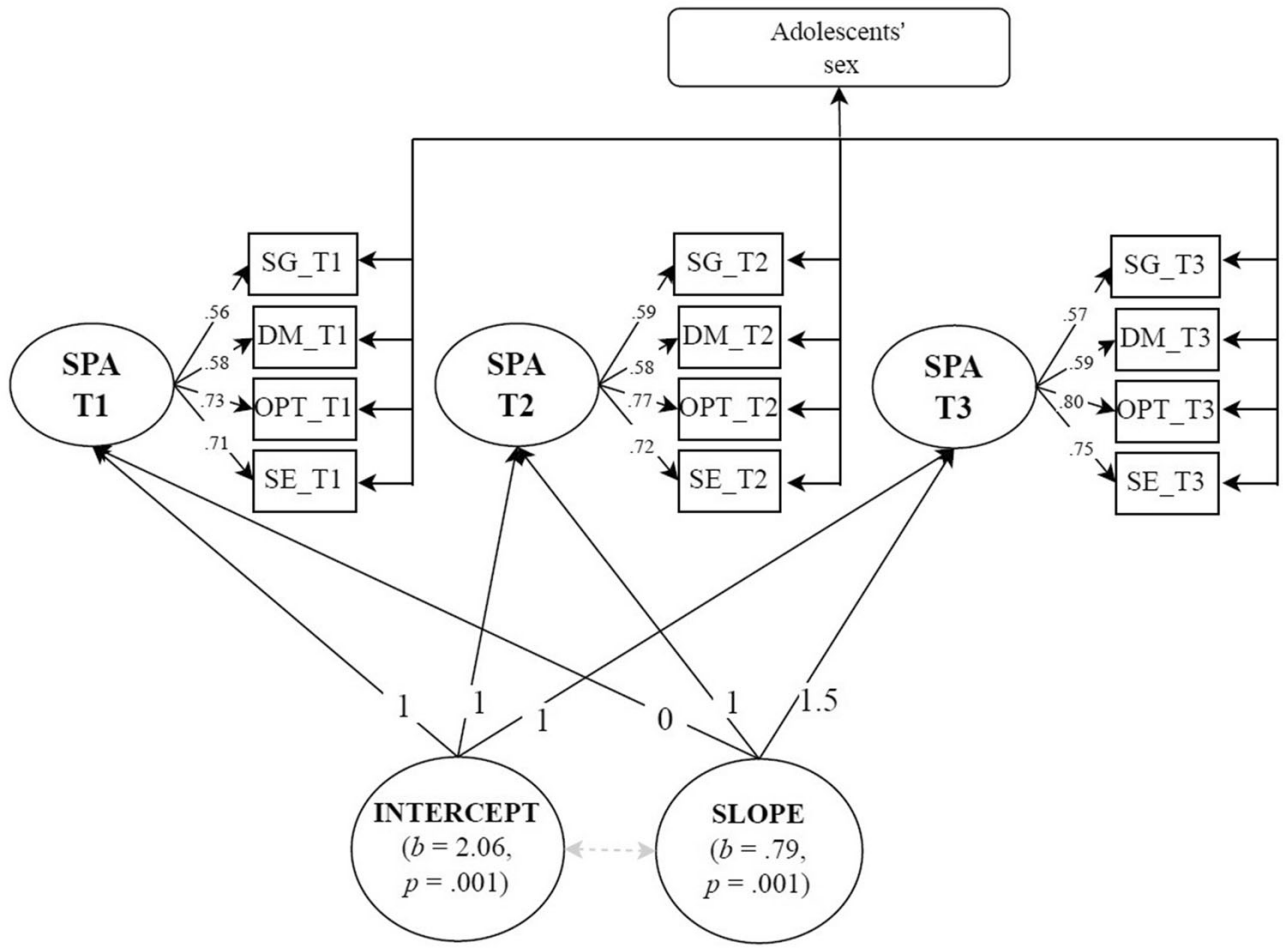
*Unconditional Latent Growth Curve (LGC) on Sense of Agency across Late Adolescence*. *Note*. SG Setting goals, DM Decision-making, OPT Optimism, SE Self-efficacy, SPA Sense of agency, T1 First assessment (baseline), T2 Second assessment (after 12 months from T1), T3 Third assessment (after six months from T2). Standardized coefficients are presented

An unconditional LGC model examined the change trajectories of adolescents’ sense of agency. Sense of agency at T1, T2, and T3 was modeled as a latent variable informed by four indicators (goal setting, decision-making, optimism, and self-efficacy). Based on the preliminary results, scalar invariance between the three assessment times was imposed. The intercept’s loadings were fixed to 1. Instead, the slope’s loadings were fixed to 0, 1, and 1.5 to account for the time gap between T1, T2, and T3. The saturated correlated approach considered the adolescents’ sex in the auxiliary variable; i.e., adolescents’ sex was correlated as all exogenous manifest variables of the LGC model. The model (see Fig. 1) estimated a mean latent intercept and slope for adolescents’ sense of agency. The results revealed an adequate fit to the data (χ^2^_(49)_ = 97.54, *p* = 0.001, CFI = 0.98, TLI = 0.97, RMSEA = 0.05, SRMR = 0.04). Adolescents reported a mean score of 2.06 (*p* = 0.001) on the sense of agency at T1, followed by a positive linear slope (*b* = 0.79, *p* = 0.001). Results also revealed significant between-subject variance at initial levels (*b* = 0.15, *p* = 0.001). On the contrary, the variance of the sense of agency rate growth was nonsignificant (*b* = 0.02, *p* = 0.448).

Following, 12 manifest variables measuring attachment to father and mother (four per each assessment point; two for the father and two for the mother) were added to the previous LGC. These variables evaluated whether variations in attachment to each parent contribute to the sense of agency at T1, T2, and T3. First, a full unconstrained model was tested. The results revealed an acceptable fit to the data (χ^2^_(181)_ = 300.66, *p* = 0.001, CFI = 0.94, TLI = 0.93, RMSEA = 0.05, SRMR = 0.07 Next, the links of attachment to father and mother on sense of agency were constrained to be equal in T1, T2, and T3. The constrained model revealed an acceptable fit to the data (χ^2^_(189)_ = 315.19, *p* = 0.001, CFI = 0.94, TLI = 0.93, RMSEA = 0.05, SRMR = 0.07), and models’ comparison indicated that the constrained model did not show significantly worse adjustment to the data (Δχ^2^_(8)_ = 10.77, *p* = 0.215). This finding suggests a stable effect of attachment to parents on adolescents’ sense of agency over time. Next, a constrained model was tested. The links of attachment to father and mother on sense of agency were fixed to be equal in T1, T2, and T3. Although the model showed an acceptable fit to the data (χ^2^_(191)_ = 321.18, *p* = 0.001, CFI = 0.94, TLI = 0.93, RMSEA = 0.05, SRMR = 0.07), models’ comparison indicated that it had a significantly worse fit to the data than the initial model (full unconstrained model) (Δχ^2^_(6)_ = 19.91, *p* = 0.003). This finding suggests differences between the effect of mothers’ and fathers’ attachment on the sense of agency over time. Based on the regression weights of the unconstrained model, the constrained model was refined by allowing different estimates to be assumed by the paths from inhibition of exploration and individuality of father and mother to the sense of agency across all assessment points. This final model did not show a significantly worse fit to the data than the unconstrained model (Δχ^2^_(5)_ = 7.15, *p* = 0.210), indicating that only inhibition of exploration had a different effect on sense of agency among parents’ sex. The results indicated that the inhibition of exploration and individuality by the mother (*B* = −0.32^1^ /−0.15/−0.15, *p* < 0.005), but not by the father, undermines sense of agency over time. In turn, the quality of the emotional bond of both mother (*B* = 0.18/0.18/0.17, *p* < 0.01) and father (*B* = 0.18/0.18/0.17, *p* < 0.01) was positively associated with sense of agency in T1, T2, and T3, respectively.

**Fig. 2:**
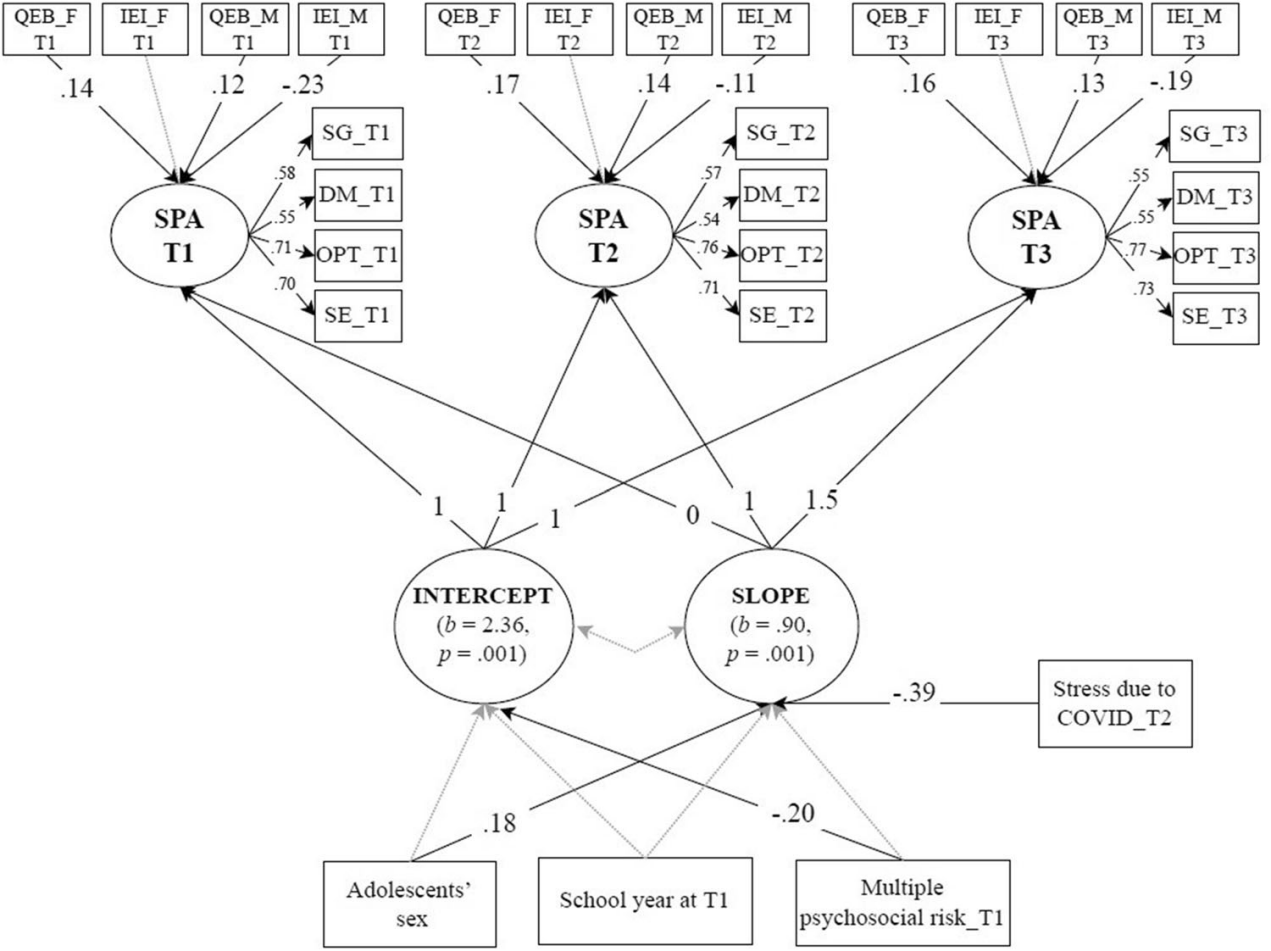
*Conditional LGC on Sense of Persona Agency: Effects of Attachment to Parents, Sex, Multiple Risk, and Pandemic-related Stress. Note*. SG Setting goals, DM Decision-making, OPT Optimism, SE Self-efficacy, SPA Sense of agency, QEB_F Quality of emotional bond with father, IEI_F Inhibition of Exploration and Individuality by father, QEB_M Quality of emotional bond with mother, IEI_M Inhibition of Exploration and Individuality by mother, T1 First assessment (baseline), T2 Second assessment (after 12 months from T1), T3 Third assessment (after six months from T2). Standardized coefficients are presented

Finally, three manifest variables (adolescent’s sex, multiple risks, and school year that adolescents attended in the baseline) were added to the constrained conditional model LGC as covariates of the intercept and slope of sense of agency. The contribution of pandemic stress in T2 on the change of the sense of agency was also tested. The model fit acceptably to the data (χ^2^_(227)_ = 375.98 *p* = 0.001, CFI = 0.93, TLI = 0.92, RMSEA = 0.05, SRMR = 0.06). Despite this acceptable fit, the nonsignificant paths with a magnitude approaching zero were trimmed. The trimmed model did not show a significantly worse adjustment to the data compared to the original model (Δχ^2^_(6)_ = 12.33, *p* = 0.055). As such, this more parsimonious model was retained. This model revealed an acceptable fit (χ^2^_(233)_ = 384.87, *p* = 0.001, CFI = 0.93, TLI = 0.92, RMSEA = 0.05, SRMR = 0.06) (Fig. 2). Results indicated that boys had a greater growth of sense of agency than girls (*B* = 0.39, *p* = 0.018). Adolescents who experienced higher multiple risks in T1 revealed lower initial levels of sense of agency (*B* = −0.12, *p* = 0.004). Further, adolescents who reported higher levels of pandemic stress in T2 revealed lower growth of sense of agency (*B* = −0.46, *p* = 0.001).

## Discussion

The literature points out that sense of agency is a dynamic and contextualized process that develops through interactions between the developing individual and the changing social context. However, studies on fluctuations in the sense of agency during adolescence, specifically during high school, where adolescents make crucial decisions about their future, still need to be made available. Documenting the stability or change in the sense of agency throughout high school education is only a first step to understanding the development of sense of agency during this crucial developmental period. Identifying predictors of stability or change establishes critical insights toward building models linking variations in adolescents’ life contexts to developing sense of agency. The current study examined changes in the trajectories of the sense of agency during the high school years. In addition, an attempt was made to understand whether attachment to both parents and whether adolescents’ sex, cumulative risk at baseline, and pandemic-related stress have related to these trajectories, controlling for the contribution of the school year that adolescents attended at baseline.

The current study established three main findings: First, sense of agency revealed a positive linear slope during high school. This growth can derive from adolescents’ biopsychosocial changes during this period (Eccles & Wigfield, 2002). The physical, cognitive, and emotional development experienced during adolescence helps young people to achieve greater autonomy from parents and to develop a more concrete notion about their values and interests, which, in turn, can strengthen their “horizon of possibilities.” Another factor that can explain these results is the growing participation of adolescents in decisions that affect their lives. Parents and teachers increasingly involve adolescents in decisions that affect them (Conger et al., 2010). As long as young people acquire more decision power, they may feel more capable of shaping their life course. These results are consistent with hypothesis 1 based on the literature suggesting that throughout adolescence, most young people tend to become increasingly active agents in their expanding social world (Thoits, 2006). These results are interesting because, despite the pandemic that affected young people’s lives, their sense of agency has revealed a growth trajectory during high school. In Portugal, between the current’s study two initial assessment waves, participants were confined for four months (March to June 2020). During this period, school activities were wholly online, and young people were deprived of face-to-face contact with figures other than the nuclear family. Despite these circumstances, there was an average increasing growth in young people’s sense of agency during this particular period.

Furthermore, although young people showed inter-individual differences in the initial values of sense of agency, there were no differences in the growth rate. These results suggest that the young people who participated in the current study all experienced growth in their sense of agency. Young people could show similar developmental growth trends even though they can vary in their biopsychosocial development (Branje, 2018). It would be valuable for future studies to analyze this development in more diverse and heterogeneous samples. Future studies should clarify whether this finding results from the characteristics of participants of this study or if they are normative and young people tend to reveal similar growth patterns in their sense of agency, whatever their starting point.

The second significant contribution of this study was to clarify the contributions of attachment to parents on sense of agency over time. The contributions of relationships with father and mother to sense of agency remained stable throughout high school. These results bring essential contributions to the literature, as they corroborate the role of attachment to adolescents’ sense of agency and clarify that this role is relatively stable over time. The perspective of relational continuity comes to mind when discussing this result. Although the content and form of parent-child relationships change as adolescents mature, the functional properties of these relationships tend to remain stable (Branje, 2018). In other words, parents’ responsiveness and availability are maintained, although the attachment behavior of adolescents reflects some distance due to the increasing need for autonomy (Branje, 2018). This relational continuity may occur in the current study, despite the significant changes in the attachment to parents. Throughout the three assessment points (preliminary exploration of data), the characteristics of relationships with father and mother that contribute to sense of agency remain the same. Despite the initial efforts to discuss these results, further studies need to clarify this issue through more extended time intervals.

Still, regarding attachment to parents, relationships with father and mother contribute differently to sense of agency over time. Results show that the quality of the emotional bond with both parents promotes a positive development of sense of agency. In contrast, the inhibition of exploration and individuality by the mother, but not the father, undermines this development. These findings expand the results from previous studies insofar as they show that the quality of the emotional bond with the father and inhibition of exploration and individuality by the mother are not only correlated concurrently with sense of agency (Nunes et al., 2022a) but also are an essential correlate for its development. Further, these findings also show that the quality of the emotional bond with the mother is crucial to shaping adolescents’ sense of agency.

Results suggest that relationships characterized by support and security with both parents throughout adolescence promote the expansion of adolescents’ perceived opportunities to shape their life course. When adolescents perceive their parents as responsive and available, they consolidate their models of inner security and trust that can reinforce their beliefs in their capacity to be active agents in their life course. However, when adolescents perceive that their mothers do not encourage their explorative initiatives and, conversely, tend to inhibit them, they feel less and less autonomous and tend to believe that a smaller range of opportunities is available to them. Adolescents who desire more autonomous and symmetrical relationships with their parents often also need more emotional closeness and open communication with their parents. Parents, in turn, can respond to their adolescents’ need for autonomy by distancing themselves from their children’s lives or demonstrating greater control and inhibition to avoid the emergence of problematic behaviors (Eccles et al., 1993). For instance, mothers, in response to their adolescents’ emerging sexuality and increased involvement with peers, tend to become more concerned and offer fewer opportunities for autonomous decision-making to their adolescent children (Eccles et al., 1993), which in turn can undermine their sense of agency. However, it should be acknowledged that these maternal behaviors may be an essential protection for some adolescents, who tend to be involved in more risky behaviors. Despite the initial insights and reflections, this issue needs further examination in future research.

Regarding the importance of the quality of emotional bonds with mothers found in the current study and not in the previous study, it is essential to clarify that the participants of the current study are, in the mean younger than adolescents in the previous study. As young people become more independent and autonomous, they may not need so much emotional support from mothers to consider themselves the authors of their life course. Despite the initial efforts to discuss these results, it would be relevant in future studies to evaluate the contribution of the quality of attachment to parents in sense of agency over a longer time to see if this developmental hypothesis is supported.

Finally, findings indicate that adolescents’ sex, cumulative risk, and pandemic-related stress affected the development of sense of agency. Concerning sex, although boys and girls did not show differences in their initial values of sense of agency, boys revealed a greater growth rate than girls over time. Boys and girls are affected differently by the demands of high school, with girls reporting significantly more concerns than boys (Rice et al., 2011). This experience, associated with the awareness of gender inequalities in Portuguese society (Brancazio, 2019), may explain the slower growth in girls’ sense of agency compared to boys. Notably, the current study’s findings expand previous evidence, clarifying that developing sense of agency is not independent of adolescents’ sex.

Regarding cumulative risk, higher levels of psychosocial risk were associated with lower initial values but not the growth rate of sense of agency. Adolescents who experienced more psychosocial risks showed lower initial values of sense of agency, but this did not affect the development of these beliefs. Results suggest that adolescents may have characteristics (e.g., personality traits) and contexts (e.g., social support) that allow them to overcome disadvantages in sense of agency throughout late adolescence. These results make essential contributions to the literature and expand the results of previous studies (Nunes et al., 2022a) insofar as they show that despite the impairment of adolescents’ sense of agency by exposure to cumulative risk, adolescents have resources capable of reducing the damaging impact of these risks as time goes. Despite these reflections, it is essential to highlight that most participants in the current study only reported a few risk factors simultaneously. It would be necessary for future studies to analyze more heterogeneous samples regarding multiple psychosocial risks. Despite the relevance of these results, recent approaches to adversity highlight that risk should be understood as a multidimensional construct composed of outside sources of adversity that the individual cannot control (hardness) and hardness variation (McLaughlin et al., 2021). It would be valuable if future studies could expand the current study’s findings, articulating this multidimensional approach to risk with young people’s sense of agency.

Moreover, pandemic-related stress undermines the growth of sense of agency. These results were expected, as many aspects of the pandemic, such as social isolation, general lockdown, and uncertainties about the disease and its evolution, led the world population to a high degree of uncertainty regarding its future (Cohen et al., 2021). It is believed that this experience had an increased impact on adolescents‘ lives who, during high school, try to develop strategies in preparation for their future. During the pandemic, adolescents had to adapt to distance learning mechanisms and “lost” the support of their peers and teachers, factors that may have triggered some doubts about their ability to achieve their self-determined goals, such as in the academic field. It would be valuable that future studies analyze the effect of pandemic stress on the trajectory of sense of agency over a longer time to effectively know the impact of the pandemic on the development of sense of agency.

This work showed an increment in adolescents’ sense of agency during high school, affected by sex, psychosocial risk, pandemic-related stress, and relationships with parents. Some limitations of previous studies were overcome, namely by analyzing longitudinal data on sense of agency and relationships with both parents and testing the contributions of the quality of these relationships across time on the development of adolescents’ sense of agency. Despite its notable strengths, this study has some limitations that must be acknowledged. First, other developmental contexts, such as peers, teachers, and the school community, are essential to sense of agency not considered in models tested in the current study. Second, the results were based on self-reported data. Future studies would be valuable to analyze these trajectories using interview data or parental reports. Third, the current study was based on a sample of adolescents in the North of Portugal, and future studies must assess the generalizability of findings across different cultural contexts. In addition, the results were based on data from adolescents who mostly lived with both parents. It may be possible that the trajectory of sense of agency over time is different between adolescents who live with both parents and youth who come from other family configurations. Additional research with more heterogeneous samples regarding family configuration would add weight to the potential implications arising from this study. It would also be valuable to analyze the effect of time-varying attachment to parents in the sense of agency from the perspective of parental figures. Further research is needed on possible bidirectional influences between a sense of agency and attachment to parents over time. The development of more studies that include new variables that explain the trajectory of sense of agency, such as basic psychological needs, relationships with peers and teachers, and belonging to schools, would allow a deeper understanding of the development of these beliefs during high school.

## Conclusion

Although the literature states that sense of agency changes over time, depending on individual, relational, and contextual factors, its development during adolescence still needs to be studied. The current study addresses this knowledge gap by analyzing the trajectories of sense of agency during high school during a global pandemic, shedding light on the contribution of time-varying relationships with parents, and the role of adolescents’ sex, cumulative risk, and pandemic-related stress on this trajectory. The findings of this study show that adolescents experience an increase in their sense of agency as they mature, with attachment to their father and mother playing a different but stable role in this growth. The sex of adolescents and the stress related to the pandemic proved to be important factors for developing sense of agency. Boys and adolescents who reported lower pandemic-related stress revealed greater growth in their sense of agency. In turn, multiple psychosocial risks undermined the initial values of the sense of agency, but this did not affect the growth rate of the sense of agency.

## Footnotes

1 In the text, non-standardized estimates are presented, while in Fig. 2, standardized estimates are presented.
